# Hierarchical ferroelectric and ferrotoroidic polarizations coexistent in nano-metamaterials

**DOI:** 10.1038/srep14653

**Published:** 2015-10-01

**Authors:** Takahiro Shimada, Le Van Lich, Koyo Nagano, Jie Wang, Takayuki Kitamura

**Affiliations:** 1 Department of Mechanical Engineering and Science, Kyoto University, Nishikyo-ku, Kyoto 615-8540, Japan; 2 Department of Engineering Mechanics, School of Aeronautics and Astronautics, Zhejiang University, Hangzhou 310027, China

## Abstract

Tailoring materials to obtain unique, or significantly enhanced material properties through rationally designed structures rather than chemical constituents is principle of metamaterial concept, which leads to the realization of remarkable optical and mechanical properties. Inspired by the recent progress in electromagnetic and mechanical metamaterials, here we introduce the concept of ferroelectric nano-metamaterials, and demonstrate through an experiment *in silico* with hierarchical nanostructures of ferroelectrics using sophisticated real-space phase-field techniques. This new concept enables variety of unusual and complex yet controllable domain patterns to be achieved, where the coexistence between hierarchical ferroelectric and ferrotoroidic polarizations establishes a new benchmark for exploration of complexity in spontaneous polarization ordering. The concept opens a novel route to effectively tailor domain configurations through the control of internal structure, facilitating access to stabilization and control of complex domain patterns that provide high potential for novel functionalities. A key design parameter to achieve such complex patterns is explored based on the parity of junctions that connect constituent nanostructures. We further highlight the variety of additional functionalities that are potentially obtained from ferroelectric nano-metamaterials, and provide promising perspectives for novel multifunctional devices. This study proposes an entirely new discipline of ferroelectric nano-metamaterials, further driving advances in metamaterials research.

Metamaterials are rapidly emerging at the frontier of science involving physics, material science, engineering and chemistry due to their exotic, tunable and sometimes even unprecedented material properties, which arise from (periodic) arrangements of rationally designed structures rather than from the intrinsic properties of the chemical constituents. Being initially intended to realize negative refraction just over a decade ago^1^,^2^, this deceptively simple but extraordinarily powerful concept then quickly covered a much broader range in the field of electromagnetics or optics, and has allowed the realization of many new and unusual optical properties, such as ultrahigh positive refractive index^3^, magnetism at optical frequencies^4^, giant circular dichroism^5^, subwavelength imaging^6^, perfect absorption^7^, and enhanced nonlinear optical properties^8^. Beyond electromagnetism, the metamaterials concept has recently been extended to classical mechanics and also to quantum mechanics^9^. Mechanical metamaterials have continued to exhibit fascinating properties, such as ultra-lightweight yet strong and recoverable metamaterials^10^, auxetic mechanical metamaterials^11^, negative compressibility and negative incremental stiffness metamaterials^12^, and an elastomechanical unfeelability cloak^13^. Therefore, further proposals and developments of new class metamaterials are promising for material science and engineering^14^.

One of the high-potential candidates to extend the concept of metamaterials is ferroelectric materials. Ferroelectric materials are at the heart of many advanced technologies, such as nonvolatile random access memory (FeRAM) devices^15^,^16^, sensors, actuators, and transducers in micro (nano) electromechanical systems (MEMS/NEMS)^17^,^18^, due to their large ferroelectricity and related electromechanical properties including a large piezoelectric response and high dielectric constant. As a characteristic of ferroelectric materials, spontaneous polarization and its arrangement (i.e., domain configuration) are crucial for determining the ferroelectric and related piezoelectric and dielectric properties. In particular, as a material approaches nanoscale dimensions, the spontaneous polarization, and thus the domain patterns, is altered due to the high surface-to-volume ratio, bringing about properties distinct from the bulk counterpart^19^. Recent studies have shown that the screening of a depolarization field in confined ferroelectrics with a size of several nanometers is enhanced through the alignment of polarization along the free surfaces, which results in the formation of closed-flux (vortex) polarization ordering, i.e., polarization vortices^20^. The vortex structure in nanoscale ferroelectrics is regarded as a toroidal order, which is distinct from the common homogeneous polarization order. The discovery of the polarization vortex has attracted considerable attention due to its scientific impact as a new class of order parameter in materials and the potential for technological applications including ultrahigh-density data storage as a novel functional device paradigm. Intensive work has thus been conducted to realize and control the vortex structure, and the formation of polarization vortices or domain structures has been demonstrated to be quite sensitive to the geometry of the nanostructures^21^. This implies the availability of an extrinsic method to tune the domain configurations and control the properties of ferroelectrics through the use of rationally designed nano-structures, i.e., the principle of the metamaterials concept. The majority of work to date on nanoscale ferroelectrics has focused on the level of individual elements, where the domain patterns adopt a single order parameter and are somewhat simple. However, to meet the ever increasing demand for further the improvement of ferroelectric devices, there is a strong requirement to stabilize and control rather complex domain patterns that possess a high possibility to offer novel functionalities. Although complex domain patterns have motivated numerous theoretical and experimental investigations^22^,^23^,^24^,^25^, they have not yet been commonly observed, and the control of such complex patterns has been elusive. Instead, a combination of several nano-ferroelectric components accommodated in a hierarchical nanostructure is predicted to create complex domain patterns due to the dual advantages of variable length-scale and dimensionality, the nano-size effect, and the interplay between polarization domains accommodated in the constituent nanostructures.

In the present study, we introduce a concept of ferroelectric nano-metamaterials, and demonstrate through conducting an experiment *in silico* with hierarchically nanostructured ferroelectrics using state-of-the-art real-space phase-field techniques based on the Landau-Devonshire theory. In this concept, the internal shape of hierarchically nanostructured ferroelectrics can be used as an additional degree of freedom to extrinsically tune the domain configurations, and thus the macroscopic properties. This new concept allows a variety of unusual and complex yet controllable domain patterns to emerge in ferroelectric nano-metamaterials. We further explore a key design feature to realize such unusual domain patterns using the parity of junctions that connect constituent nanostructures in the metamaterials.

To illustrate the idea of ferroelectric nano-metamaterials, 11 two-dimensional (2D) nano-specimens of ferroelectric PbTiO_3_ are designed based on architectures of a completed set of 2D Archimedean lattices (ALs) (see Fig. 1), then a simulation is performed to test the formation of spontaneous polarization in such structures at the room temperature. ALs, first introduced by Kepler in 1619^26^, are defined as edge-to-edge tiling of a plane with regular polygons such that all vertices are of the same type. There are 11 ALs, in which three consist of a specific polygon (squares, triangles, or hexagons, as shown in Fig. 1a–c, respectively), and eight require the combination of two or more different polygons (Fig. 1d–k), which gives rise to further complicated internal structures. Ubiquitous members of the ALs are the square, honeycomb, triangular, and kagome lattices, while more exotic lattices are the CaVO, star, SrCuBO, bounce, trellis, maple-leaf, and SHD lattices^27^. The structural diversity and intriguing characteristics of ALs provide an ideal playground for a systematic study of the interplay between nano-metamaterials structures and ferroelectricity.

Taking into account the strong effect of the depolarization field, the dimensions of all lattices in the specimens are set in a range from several to tens of nanometers. Therefore, the thickness of specimens and the width of the lattices are prepared at 4 and 6 nm, respectively. To access the intrinsic role of the nano-metamaterial structure on the domain configurations, free-standing specimens with the same filling fraction of 30% are investigated. The periodicity is taken in the *x*_1_ and *x*_2_ directions. The specimens consist of a single crystal of ferroelectric PbTiO_3_, where the [100], [010], and [001] axes correspond to the *x*_1_, *x*_2_, and *x*_3_ directions, respectively.

In the present study, the formation of spontaneous polarization in free-standing nano-metamaterials specimens at room temperature is carefully tested using sophisticated real-space phase-field techniques based on the Landau-Devonshire theory, which explicitly includes the depolarization effect and non-trivial electro-elastic coupling (see Supplementary Material). To realize bare free surfaces of the specimens, the electrical boundary conditions on all surfaces are set to be open-circuited, i.e., **D**·**n** = 0, so that the depolarizing fields from the polar surfaces are explicitly taken into account. Note that our preliminary analysis showed that there is no difference in polarization domain structures under open-circuited and general electrostatic boundary conditions (as shown in Supplementary Figure S1 and S2). The open-circuited boundary condition is employed in consideration of computing resources due to the complex geometry of AL structures. Testing is started by the introduction of an initial state with random fluctuations of polarization around zero, which recognizes the paraelectric state of ferroelectric nano-metamaterials. At room temperature, evolution of the polarization field toward its thermodynamic equilibrium distribution is driven by the decrease of total free energy in the ferroelectric system, which is obtained by numerically solving the time-dependent Ginzburg-Landau equation (see Supplementary Material). The domain patterns are then stably formed when no significant change in the polarization can be observed. The properties of PbTiO_3_ have been described in previous study^28^.

Figure 2a–c show the spontaneous polarization distribution at the thermodynamic equilibrium state in square, honeycomb, and triangular specimens, respectively. A strong confinement in the *x*_3_ direction of the nano-metamaterials results in purely in-plane polarization, which is consistent with the experimental observations^22^,^23^,^24^,^29^,^30^ and simulated predictions^31^,^32^ for ferroelectric lamellae. The contour color indicates the angle *θ* between the polarization vector ***P*** and the *x*_1_ direction. Figure 2 shows that the polarizations align along the lattices and thus form a normal rectilinear domain accommodated in each lattice. Such a single domain configuration causes a decrease in the electrostatic interaction energy through elimination or reduction of the depolarization fields at free surfaces. Thus, the common single domain is an energetically favorable domain configuration that appears in the lattices of nano-metamaterials. Unlike a conventional configurations of random rectilinear domains in bulk ferroelectric materials or regular arrays of stripe domains in ferroelectric thin films, the single domains in the square, honeycomb, and triangular nano-metamaterials connect to each other by gradually rotating the orientation of the local polarization vectors at the lattice junctions to form a continuous flow pattern. Such continuous flow represents novel polarization patterns that are characteristic for the square, honeycomb, and triangular specimens.

Figure 3a–c depict domain configurations at the thermodynamic equilibrium state in the CaVO, star, and SrCuBO specimens, respectively. Single domains are also formed in most lattices of the specimens and connect themselves through alteration of the local polarization orientations at the junctions. Besides the rectilinear domains, the polarizations in some lattices terminate the continuous flow and then curl their orientation to form clockwise and/or counter-clockwise microvortices. Such unusual domain configurations with ferrotoroidic polarization due to the strong depolarization field have been recently reported in various isolated ferroelectric nanostructures, such as nanodots^20^, nanowires^33^, and nanobars^34^,^35^. The local microvortex is characterized by a toroidal moment, i.e., 
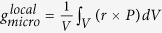
36, where *r* is the position vector, *V* is the ferroelectric volume that the vortex occupies, and *P* is the spontaneous polarization vector. In the present study, the toroidal moment, 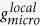, is determined to be equivalent to 0.154 e/Å, which is similar to that obtained for a nanodot^37^. This shows that a microvortex in a ferroelectric nano-metamaterial is intrinsically similar to the vortex in a nanodot. On the other hand, the most valuable feature that emerges in the CaVO, star, and SrCuBO specimens is the coexistence of vortices and rectilinear polarizations, which are characterized by two different order parameters, i.e., ferrotoroidic and ferroelectric order parameters, respectively. The coexistence permits a cross-coupling between two order parameters that appear in the same ferroelectric nanostructure, and makes it possible to tailor the toroidal moments by a homogeneous electric field. As a result of the coexistence, spontaneous polarization forms an unusual pattern with a maze-like polar structure. The coexistence of the ferroelectric and ferrotoroidic domains, and the resulting maze-like polarization pattern are characteristics of the CaVO, star, and SrCuBO specimens. Since the polarization found a preferred path to continuously connect through the maze-like pattern, this would be particularly useful for next-generation of chirality logic devices^38^. Furthermore, the complexity of the maze-like pattern may bring about an unusual domain switching process, such as multi-step switching process that potentially leads to interesting multi-state devices.

Figure 4g,h show that the continuous flow pattern of polarization also appears in the kagome and bounce specimens at the thermodynamic equilibrium state. However, an intriguing characteristic that is distinct from the continuous flow pattern in the square, honeycomb, and triangular specimens (shown in Fig. 2) is a nesting of flux-closure configurations of polarization at the larger mesoscopic scale, which occupies a complete loop of several neighboring single domains, to form a mesovortex. Staggered chirality of alternating clockwise and counter-clockwise mesovortices appear in the kagome specimen, while necklace-like chains of mesovortices are observed in the bounce specimen. The mesovortices in the kagome and bounce specimens are characteristically different from that observed in isolated nanostructures such as nanodots and nanowires, due to the absence of a physical vortex core, which facilitates elimination of the energetic vortex core. The local toroidal moment of the mesovortex is defined in a similar manner to that of the microvortex: 
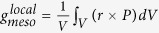
, where *V* is the volume of the ferroelectric media within the mesovortex. The toroidal moment of the mesovortex is determined to be 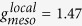 e/Å, which is approximately ten times higher than that for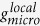. Therefore, the mesovortex is distinct from the microvortex not only by the absence of physical vortex core and its size but also by the magnitude of its toroidal moment. The appearance of mesovortices in a continuous flow pattern of polarization such as that characterized for the kagome and bounce specimens results in an intriguing and unusual polarization pattern. Additionally, the absence of physical vortex core and large toroidal moment may possess high piezotoroidic effect^39^, which is a new kind of electromechanical coupling of strain/stress and toroidal moment, providing a new additional functionality that can be exploited for electromechanical devices.

Figure 5a–c feature striking characteristics in the polarization patterns in the respective trellis, maple-leaf, and SHD specimens at the thermodynamic equilibrium state. Here, the polarization vortex occurs simultaneously at two different length scales within the single structure of the nano-metamaterial, thereby exhibiting hierarchical vortices. To the best of our knowledge, such hierarchical vortex patterns are unprecedented for ferroelectric or ferromagnetic vortices. As a result of the coexistence of hierarchical rectilinear, micro- and mesovortex polarizations, unusual and complicated patterns emerge in the trellis, maple-leaf and SHD specimens. Pairs of stable clockwise and counter-clockwise mesovortices appear surrounded by rectilinear domains and the microvortices in the trellis and maple-leaf specimens. A two-lobe-shaped mesovortex that not only occupies a large area of three adjacent polygons but also traps two micro-vortices inside it emerges in the SHD specimen to exhibit a vortex-in-vortex configuration, which is reminiscent of fractal behavior. The discovery of the nested dual-scale vortex states coexistent with rectilinear domains establishes a new benchmark in our exploration of complexity in the spontaneous ordering of electrical dipoles, which have the potential to produce unique functionalities and tunability through the coupling of two different order parameters, similar to multiferroics.

The surprising wealth of polarization patterns shown in Figs 2, 3, 4, 5 is interestingly a consequence of a well texture to form characterized polarization patterns from existing polarization domains, i.e., single domains and polarization vortices, rather than a haphazard combination of them. This suggests that the connectivity of lattices at the junctions is crucial to understand the mechanisms that lead to the formation of unusual polarization patterns in ferroelectric nano-metamaterials. Only one type of junction is structurally permitted for a given AL, which is characterized by the odd-numbered (odd-junction) and even-numbered (even-junction) binding lattices. From an energetic perspective, the polarization in ferroelectric nano-metamaterials would continue to join together with a head-to-tail arrangement due to the long-range electrostatic interaction in order to reduce the electrostatic energy, which drives the connection of the polarization domains in neighboring lattices. A careful observation of the polarization configurations presented in Figs 2, 3, 4, 5 reveals that a connection preferably involves two single domains that are jointed at the junction to maintain the head-to-tail arrangement (except the honeycomb specimen, which consists of connections of 3 single domains due to the 3-fold symmetry junction). As a result, a continuous flow pattern of polarization is spontaneously formed in the specimens that consist of even-junctions (Figs 2 and 4), where every single domain in the lattices establishes a connection to an adjacent domain. In contrast, the connection of polarization becomes more complex in the specimens with odd-junctions (Figs 3 and 5) because the polarization domains have to emulate each other to establish a connection, which leads to the termination of polarization in one lattice. The polarizations in such an uncoupled lattice would no longer be favored with a rectilinear domain, but the orientation would instead curl to produce a vortex domain so that the internal depolarization is reduced, which arises from the termination of continuous flow. Therefore, unlike isolated nanodots or nanobars, the mechanisms that leads to the formation of microvortices in ferroelectric nano-metamaterials is caused not only by free surface depolarization but also by internal depolarization and elastic interaction. The formation of microvortices in a structure with odd-junctions reveals the underlying reason for the stable coexistence of the rectilinear domains and polarization vortices in ferroelectric nano-metamaterials, which is difficult to achieve in simple isolated ferroelectric nanostructures. Thus, the junctions of lattices in ferroelectric nano-metamaterials are the key design features of our proposed concept.

Given the intriguing features of polarization patterns in ferroelectric nano-metamaterials, we proceed by considering the macroscopic ferroelectric properties to provide a comprehensive picture of the interrelationships between nano-metamaterial structures, polarization patterns, and the global behavior in ferroelectric nano-metamaterials. Table 1 summarizes the average polarization, |*P*_ave_|, and its orientation, *θ,* toward the *x*_1_ direction in ferroelectric nano-metamaterials, and the density of vortices (micro- and mesovortices), which is defined by the number of vortices per the number of lattices in the ferroelectric nano-metamaterials. The average polarization at all the sites is determined as 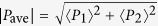, in which 
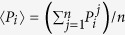
, and *n* is the total number of the nodes of simulated models. |*P*_ave_| and *θ*, which characterize the global polarization, cover a wide range of magnitudes, depending on the structure of the nano-metamaterial. This indicates that the control of the shape of ferroelectric nano-metamaterials can be used to further tailor the magnitude and direction of global spontaneous polarization. The zero net polarization shown in Table 1 is attributed to the high density of vortices, which is significantly dependent on the structures of the junctions and the shape of the ferroelectric nano-metamaterials. On the other hand, several equivalent states of polarization patterns, which are depicted in Figure S3 (Supplementary Material), can be realized due to the symmetry of the nano-metamaterials structures. The number of states, *n*, is also listed in Table 1 and its maximum can be achieved as equivalent to the symmetry order of the nano-metamaterial structure. Thus, control of the ferroelectric nano-metamaterial shape provides a novel way to control the global ferroelectric characteristics, including the magnitude and direction of macroscopic polarization, the density of vortices, and the number of stable states of domain patterns.

Our proposal of this ferroelectric nano-metamaterials concept provides a fundamentally new way to achieve and manipulate a rich diversity of complex domain patterns. The present results further indicate the possibility to obtain novel functionalities that could be particularly useful for the development of ferroelectric devices. Firstly, the mechanical and electrical responses of ferroelectric nano-metamaterials to a uniform external field would be inhomogeneous due to their diversity of lattice directions and polarization domains. This suggests an alternative strategy to efficiently tailor the piezoelectric properties through deliberate control of the internal shape of the ferroelectric nano-metamaterial, which is distinct from conventional methods such as doping^40^, and domain wall engineering^41^. Secondly, the presence of multiple equivalent stable states could realize the possibility to switch from one stable state to another by application of an external electrical field with a particular direction, giving rise to highly tunable ferroelectricity that would be appealing for multistate logic. Thirdly, the presence of mesovortices along with the ability of local polarization switching at lattices suggests that the toroidal moment of mesovorties could be strongly affected and even switched by homogeneous electric fields. The switching of polarization vortices by using the conventional homogeneous electric field suggests an alternative and practical way to control the toroidal moment, which is easier than the recently proposed use of a curled electric field^42^. Finally, the coexistence of ferroelectric and ferrotoroidic domains in ferroelectric nano-metamaterials may give rise to a cross-coupling between two order parameters due to the intrinsic relationship between ferroelectricity and ferrotoroidicity, which would enable control of the chirality of the vortex domain structure by controlling the direction of the adjacent polar domain. The coupling interaction therefore provides an additional degree of freedom in the design of multifunctional devices, and possesses a great potential for unconventional functionalities. Significant advances in micro- and nanoscale fabrication techniques have recently achieved hierarchical nanostructures of ferroelectric materials^43^, thereby providing potential for the realization of such design possibilities. However, further research is required to conduct detailed investigation into the possibilities for ferroelectric nano-metamaterials.

In summary, we have proposed a new concept of ferroelectric nano-metamaterials and demonstrated that this new concept enables the discovery of a variety of unusual and complex yet controllable domain patterns, extending our knowledge on the possible domain patterns that can form spontaneously in ferroelectric materials. The coexistence between hierarchical ferroelectric and ferrotoroidic polarizations establishes a new benchmark in our exploration of complexity in the spontaneous ordering of electrical dipoles, facilitating access to the stabilization and control of complex domain structures. Tailoring the domain configuration through control of the ferroelectric nano-metamaterial structure paves a novel way for manipulation of the properties of ferroelectric materials, including not only the macroscopic properties such as macroscopic polarization and piezoelectric response but also ferrotoroidicity and the numbers of stable states of polarization. The present study proposes an entirely new discipline of ferroelectric nano-metamaterials that can be compared to those of electromagnetic metamaterials and mechanical metamaterials, further driving the advance of metamaterials research. The present work opens a rich avenue in the field of metamaterials, which is expected to evolve fruitful ideas to achieve even more fascinating properties of ferroelectric materials. The proposed concept may stimulate future experimentation and simulation efforts to explore and realize novel functionalities in ferroelectric nano-metamaterials. In addition, this new concept can be extended to the other ferroic and multi-ferroic systems.

## Additional Information

**How to cite this article**: Shimada, T. *et al.* Hierarchical ferroelectric and ferrotoroidic polarizations coexistent in nano-metamaterials. *Sci. Rep.* **5**, 14653; doi: 10.1038/srep14653 (2015).

## Supplementary Material

Supplementary Information

## Figures and Tables

**Figure 1:**
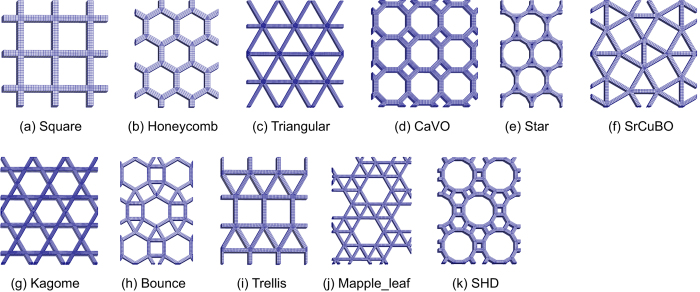
Ferroelectric nano-metamaterial specimens with 2D Archimedean lattice structures.

**Figure 2:**
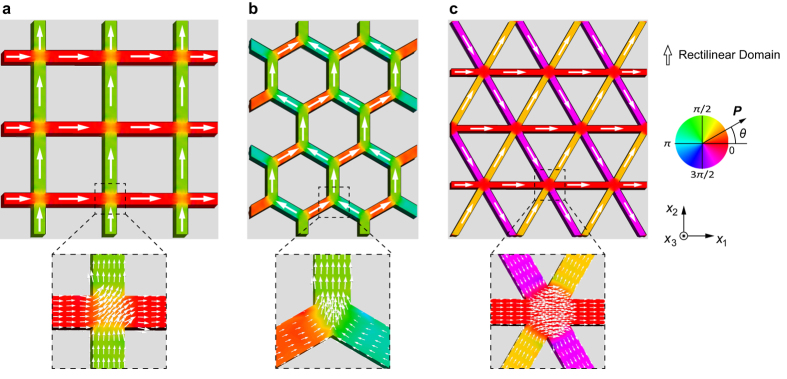
Polarization distribution in ferroelectric nano-metamaterials of (**a**) square, (**b**) honeycomb, and (**c**) triangular specimens. The polarization patterns are characterized by the continuous flow of polarization. The contours indicate the angle between the polarization vector *P* and the [100] direction.

**Figure 3:**
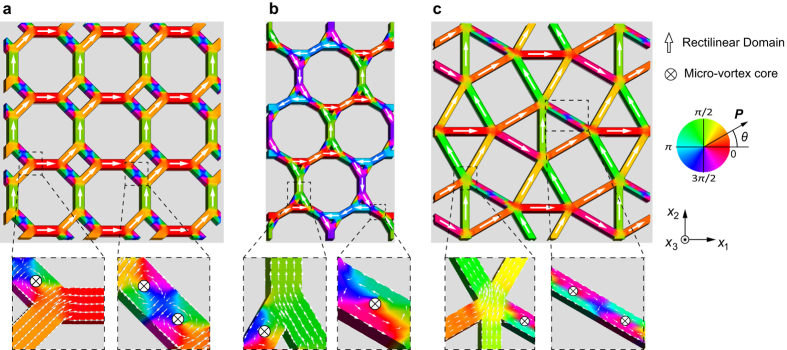
Polarization distributions in ferroelectric nano-metamaterials of (**a**) CaVO, (**b**) star, and (**c**) SrCuBo specimens. The polarization is characterized by the coexistence of rectilinear and microvortex polarizations. The contours indicate the angle between the polarization vector *P* and the [100] direction.

**Figure 4:**
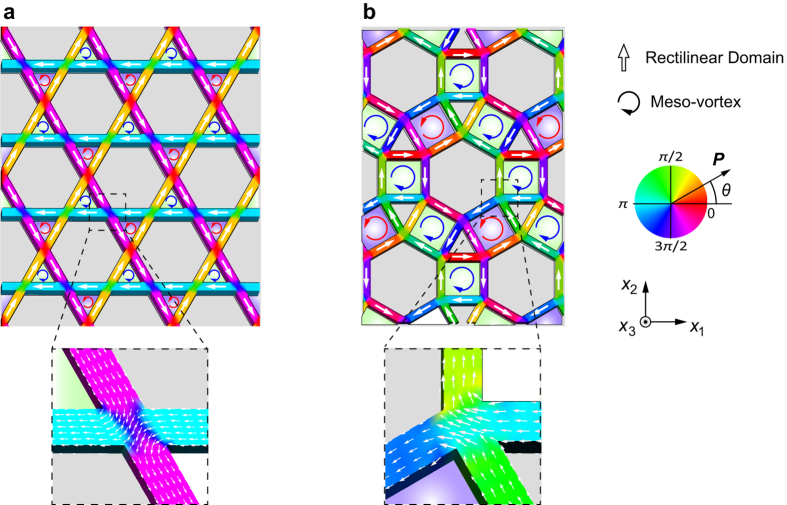
Polarization distributions in ferroelectric nano-metamaterials of (**a**) kagome and (**b**) bounce specimens. The polarization patterns are characterized by mesovortex polarization appearing in a continuous flow configuration. The contours indicate the angle between the polarization vector *P* and the [100] direction.

**Figure 5:**
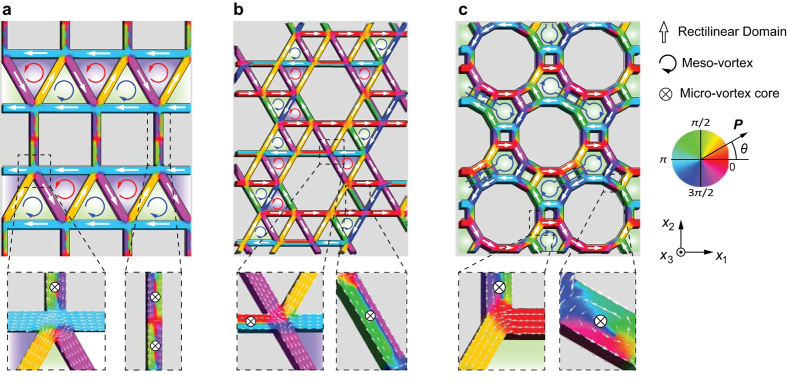
Polarization distributions in ferroelectric nano-metamaterials of (**a**) trellis, (**b**) maple-leaf, and (**c**) SHD specimens. The polarization patterns are characterized by the coexistence of hierarchical vortex and rectilinear polarizations. The contours indicate the angle between the polarization vector *P* and the [100] direction.

**Table 1:** Structural and global ferroelectric characteristics of ferroelectric nano-metamaterials.

	Structural characteristics		Global ferroelectric characteristics			
	Number of lattices at junction	Structural symmetry order	Average polarization, P_ave_ (C/m^2^)	Direction of P_ave_ to the *x*_1_ axis, *θ* (degree)	Density of vortices	Number of states,*n*
Square	4	4	0.534	45	0	4
Honeycomb	3	6	0.432	90	0	6
Triangular	6	6	0.481	45	0	6
CaVO	3	4	0	0	2/3	4
Star	3	6	0	0	6/19	3
SrCuBo	5	4	0.407	78	1/10	4
Kagome	4	6	0	0	1/3	2
Bounce	4	6	0	0	1/4	3
Trellis	5	2	0	0	4/5	2
Mapple-leaf	5	6	0.178	72	4/15	6
SHD	3	6	0	0	1/3	6
