# Supplementary material for: Hierarchical ferroelectric and ferrotoroidic polarizations coexistent in nano-metamaterials
Source: Sci Rep. 2015 Oct 1;5:14653. doi: 10.1038/srep14653 (PMC4589792; doi:10.1038/srep14653)
Supplement: Supplementary Information [file srep14653-s1.pdf]

Supplementary Material for

Hierarchical ferroelectric and ferrotoroidic polarizations coexisted in nano-metamaterials

Takahiro Shimada, Le Van Lich, Koyo Nagano, Jie Wang, and Takayuki Kitamura

Experimental Section

The spontaneous polarization patterns of ferroelectric nano-metamaterials are studied by using the real-space phase-field model based on the Ginzburg-Landau theory. In the phase-field model of ferroelectric materials, polarization vector, $\boldsymbol{P} = (P_1, P_2, P_3)$, is taken as the order parameter to describe free energies of the ferroelectric system. The total free energy of the ferroelectric system, F, can be described by [1]

$$F = \int_V f dV = \int_V \left(f_{Land} + f_{elas} + f_{coup} + f_{grad} + f_{elec} \right) dV \tag{1.1}$$

where f_{Land}, f_{elas}, f_{coup}, f_{grad}, and f_{elec} denote the Landau energy density, the elastic energy density, the coupling energy density, the gradient energy density, and the electrostatic energy density, respectively. V is the entire volume of the ferroelectric system.

The Landau energy density is expressed by a six-order polynomial of the spontaneous polarization as [2]

$$\begin{aligned} f_{Land} = {} & \alpha_1\left(P_1^2 + P_2^2 + P_3^2\right) + \alpha_{11}\left(P_1^4 + P_2^4 + P_3^4\right) \\ & + \alpha_{12}\left(P_1^2 P_2^2 + P_2^2 P_3^2 + P_3^2 P_1^2\right) + \alpha_{111}\left(P_1^6 + P_2^6 + P_3^6\right) \\ & + \alpha_{112}\left[P_1^4\left(P_2^2 + P_3^2\right) + P_2^4\left(P_1^2 + P_3^2\right) + P_3^4\left(P_1^2 + P_2^2\right)\right] \\ & + \alpha_{123} P_1^2 P_2^2 P_3^2 \end{aligned} \tag{1.2}$$

where $\alpha_1 = (T - T_0) / 2\kappa_0 C_0$ is the dielectric stiffness, α_{11}, α_{12}, α_{111}, α_{112}, and α_{123} are higher order-stiffness coefficients, T and T_0 denote the temperature and the Curie-Weiss temperature, respectively, C_0 denotes the Curie constant, and κ_0 denotes the dielectric constant of vacuum. The strain energy density is given by

$$f_{elas} = \frac{1}{2} c_{11}\left(\varepsilon_{11}^2 + \varepsilon_{22}^2 + \varepsilon_{33}^2\right) + c_{12}\left(\varepsilon_{11}\varepsilon_{22} + \varepsilon_{22}\varepsilon_{33} + \varepsilon_{33}\varepsilon_{11}\right) + 2c_{44}\left(\varepsilon_{12}^2 + \varepsilon_{23}^2 + \varepsilon_{31}^2\right) \tag{1.3}$$

where c_{11}, c_{12}, and c_{44} are the elastic constants. The coupling energy density is given by

$$\begin{aligned} f_{coup} = &-q_{11}\left(\varepsilon_{11}P_1^2 + \varepsilon_{22}P_2^2 + \varepsilon_{33}P_3^2\right) - q_{12}\left[\varepsilon_{11}\left(P_2^2 + P_3^2\right) + \varepsilon_{22}\left(P_3^2 + P_1^2\right) + \varepsilon_{33}\left(P_1^2 + P_2^2\right)\right] \\ &- 2q_{44}\left(\varepsilon_{11}P_1P_2 + \varepsilon_{13}P_1P_3 + \varepsilon_{23}P_2P_3\right) \end{aligned} \tag{1.4}$$

where q_{11}, q_{12}, and q_{44} are electrostrictive coefficients. The gradient energy density is given by

$$\begin{aligned} f_{grad} = &\frac{1}{2} G_{11}\left(P_{1,1}^2 + P_{2,2}^2 + P_{3,3}^2\right) + G_{12}\left(P_{1,1}P_{2,2} + P_{2,2}P_{3,3} + P_{3,3}P_{1,1}\right) \\ &+ \frac{1}{2} G_{44}\left[\left(P_{1,2} + P_{2,1}\right)^2 + \left(P_{2,3} + P_{3,2}\right)^2 + \left(P_{1,3} + P_{3,1}\right)^2\right] \\ &+ \frac{1}{2} G'_{44}\left[\left(P_{1,2} - P_{2,1}\right)^2 + \left(P_{2,3} - P_{3,2}\right)^2 + \left(P_{1,3} - P_{3,1}\right)^2\right] \end{aligned} \tag{1.5}$$

where G_{11}, G_{12}, G_{44}, and G'_{44} are the gradient coefficients. The gradient energy is the penalty for the spatially inhomogeneous polarization. The electrostatic energy density, which is obtained through Legendre transformation, is given as

$$f_{elec} = -\frac{1}{2}\kappa_0\left(E_1^2 + E_2^2 + E_3^2\right) - E_1P_1 - E_2P_2 - E_3P_3 \tag{1.6}$$

The temporal evolution for polarization or domain structure is calculated by the time-dependent Ginzburg-Landau equation

$$\frac{\partial P_i(\boldsymbol{r},t)}{\partial t} = -L\frac{\delta F}{\delta P_i(\boldsymbol{r},t)} \tag{1.7}$$

where t represents time, L is the kinetic coefficient related to the domain mobility, $\delta F/\delta P_i\,(\boldsymbol{r}, t)$ denotes the thermodynamic driving force for polarization evolution, and $\boldsymbol{r}$ is the spatial vector. In addition to the time-dependent Ginzburg-Landau equation, the following mechanical equilibrium equation

$$\frac{\partial}{\partial x_j}\left(\frac{\partial f}{\partial \varepsilon_{ij}}\right) = 0 \tag{1.8}$$

and Maxwell's (or Gauss) equation

$$\frac{\partial}{\partial x_i}\left(-\frac{\partial f}{\partial E_i}\right) = 0 \tag{1.9}$$

must be satisfied for charge and body force free ferroelectric materials simultaneously.

Using the variation or principal of virtual work, the governing Equations (7)-(9) are expressed in the integral form (or weak form) as [3]

$$\int_V \left\{ \frac{\partial f}{\partial \varepsilon_{ij}} \delta \varepsilon_{ij} + \frac{\partial f}{\partial E_i} \delta E_i + \frac{1}{L} \frac{\partial P_i}{\partial t} \delta P_i + \frac{\partial f}{\partial P_i} \delta P_i - \left(\frac{\partial f}{\partial P_{i,j}} \right) \delta P_{i,j} \right\} dv$$

$$= \int_S \{ t_i \delta u_i - w d\varphi + \pi_i \delta P_i \} dA, \tag{1.10}$$

where t_i is the surface traction, w denotes surface charge, and $\pi_i = \frac{\partial f}{\partial P_{i,j}} n_j$ represents the surface gradient flux. A nonlinear finite element method [1] is employed to solve Eq. (1.10), which is suitable for the arbitrary geometry of ferroelectric nano-mesomaterials.

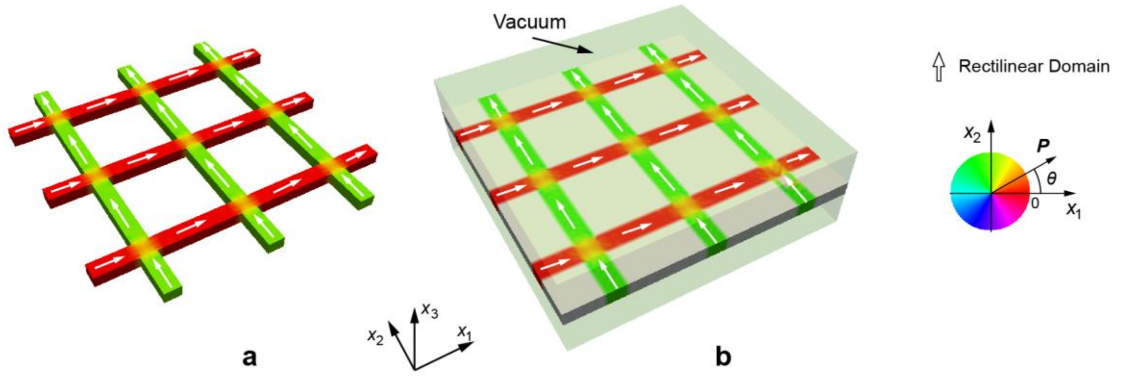

Figure S1. Polarization domain structures in the square model under (**a**) open-circuited boundary condition and (**b**) general electrostatic boundary condition.

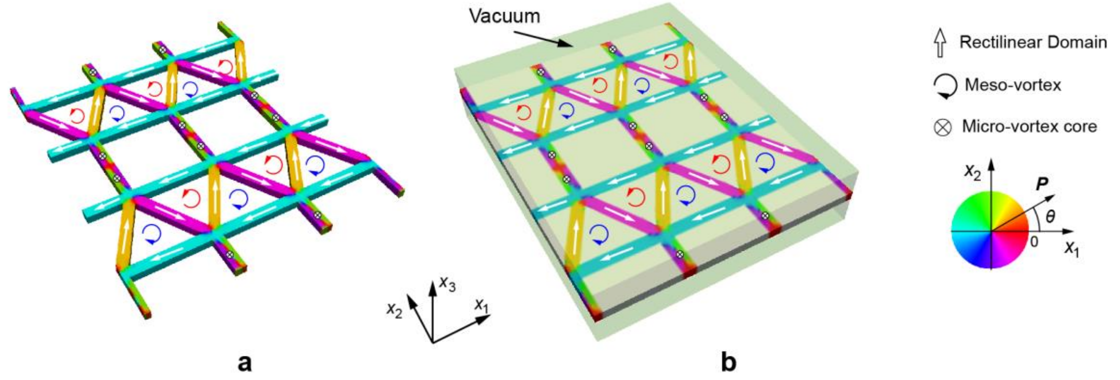

Figure S2. Polarization domain structures in the trellis model under (**a**) open-circuited boundary condition and (**b**) general electrostatic boundary condition.

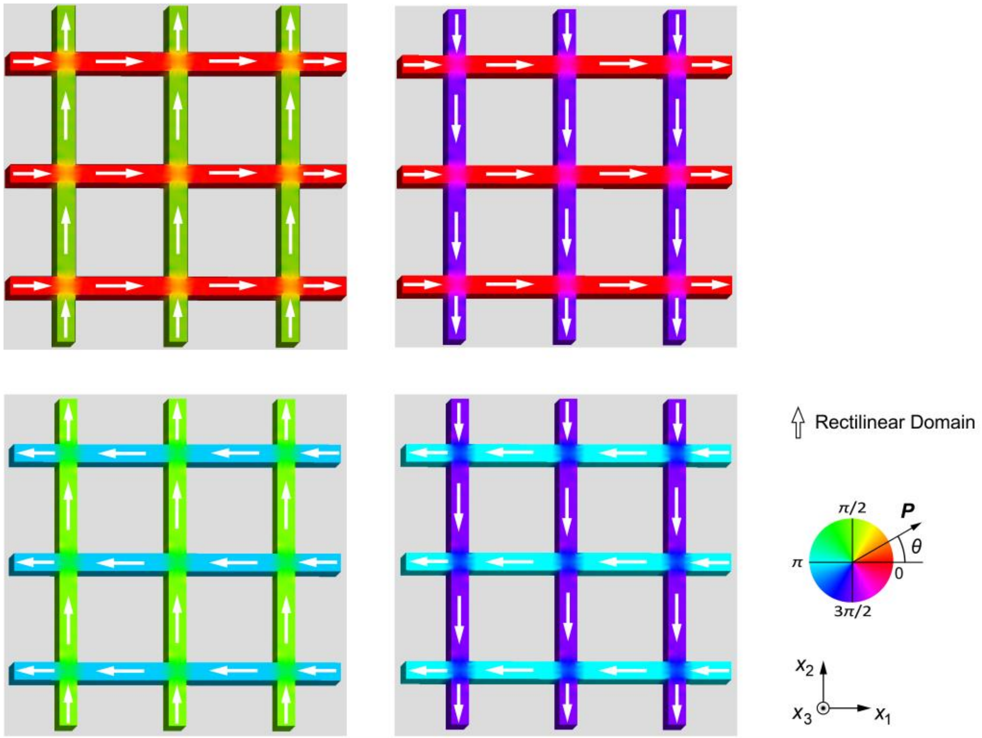

Figure S3(a). 4-equivalent stable states of the Square specimens.

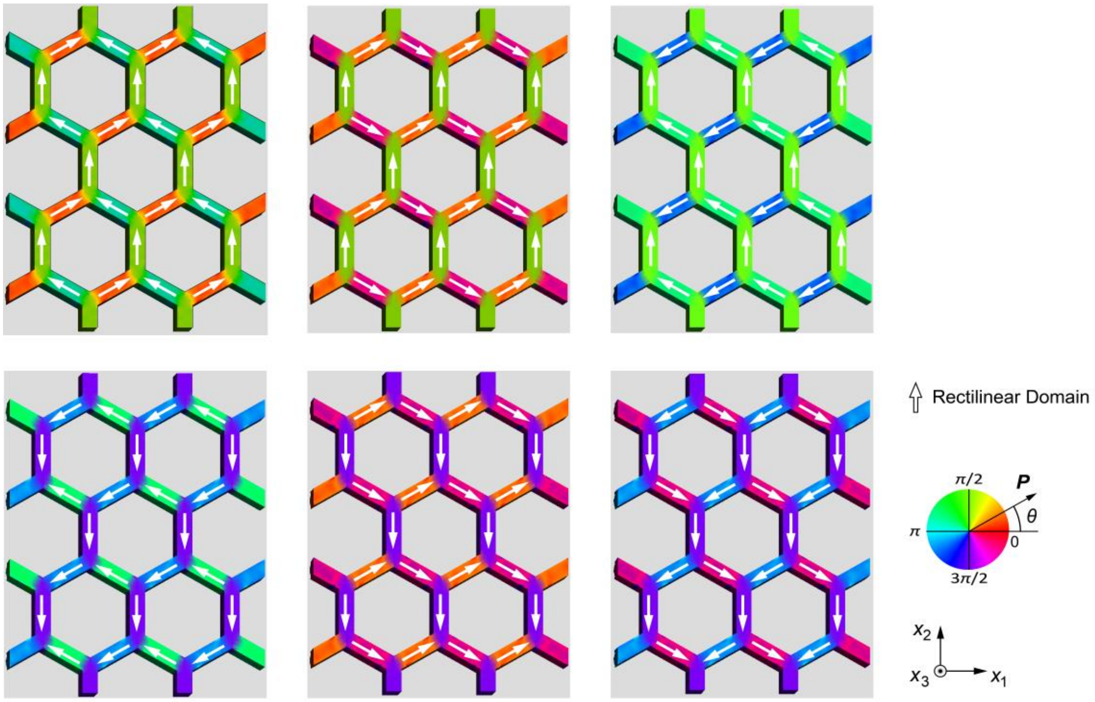

Figure S3(b). 6-equivalent stable states of the Honeycomb specimen.

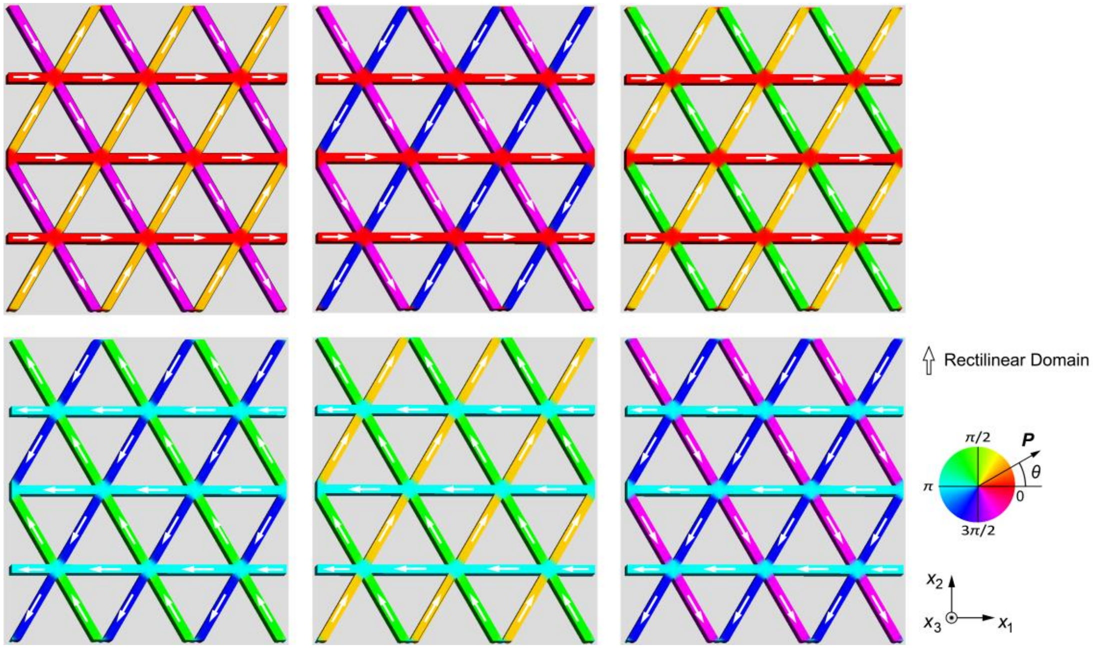

Figure S3(c). 6-equivalent stable states of the Triangular specimen.

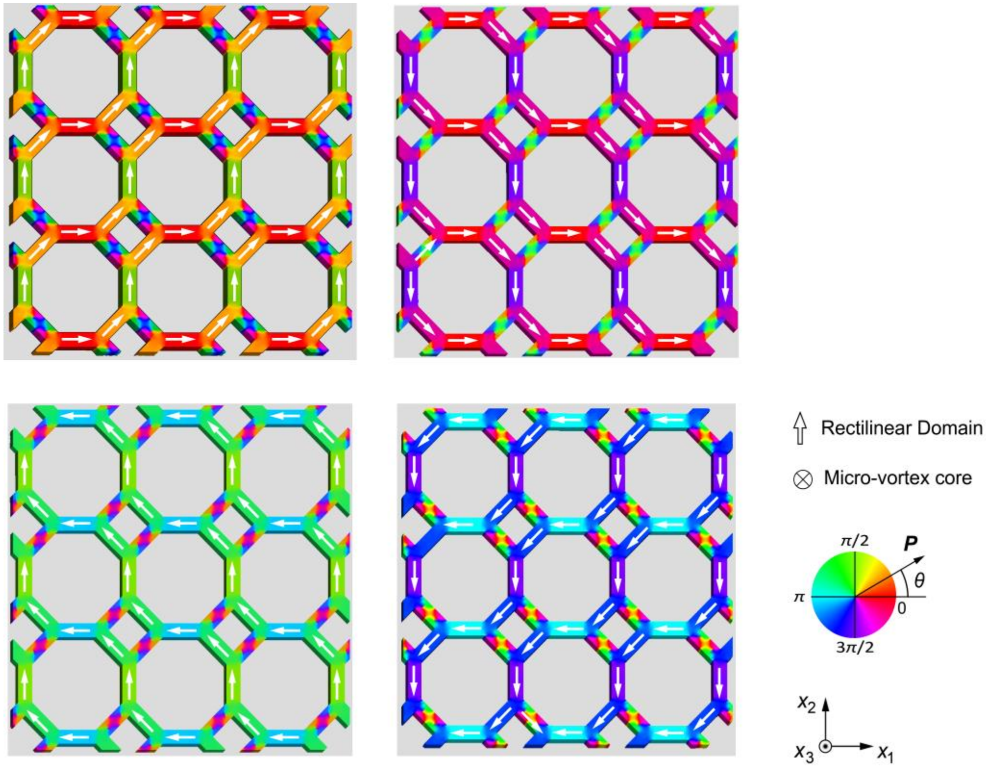

Figure S3(d). 4-equivalent stable states of the CaVO specimen.

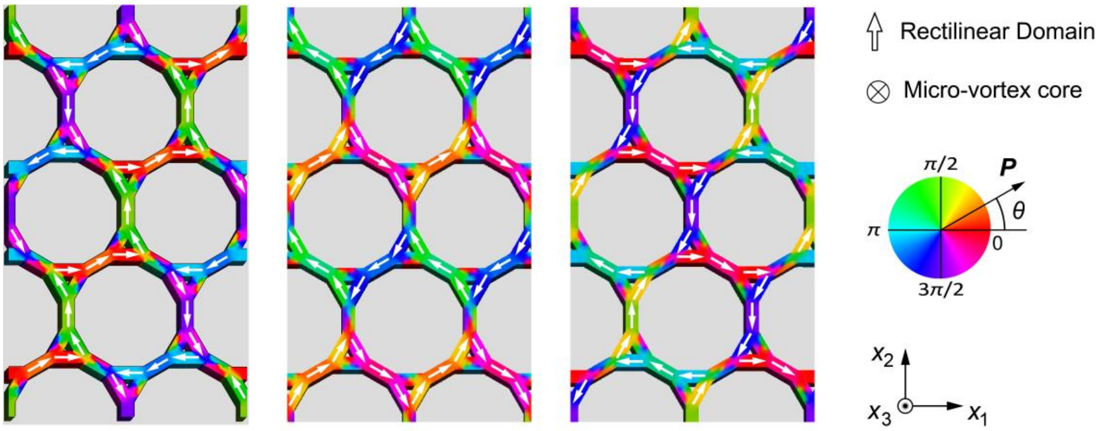

Figure S3(e). 3-equivalent stable states of the Star specimen.

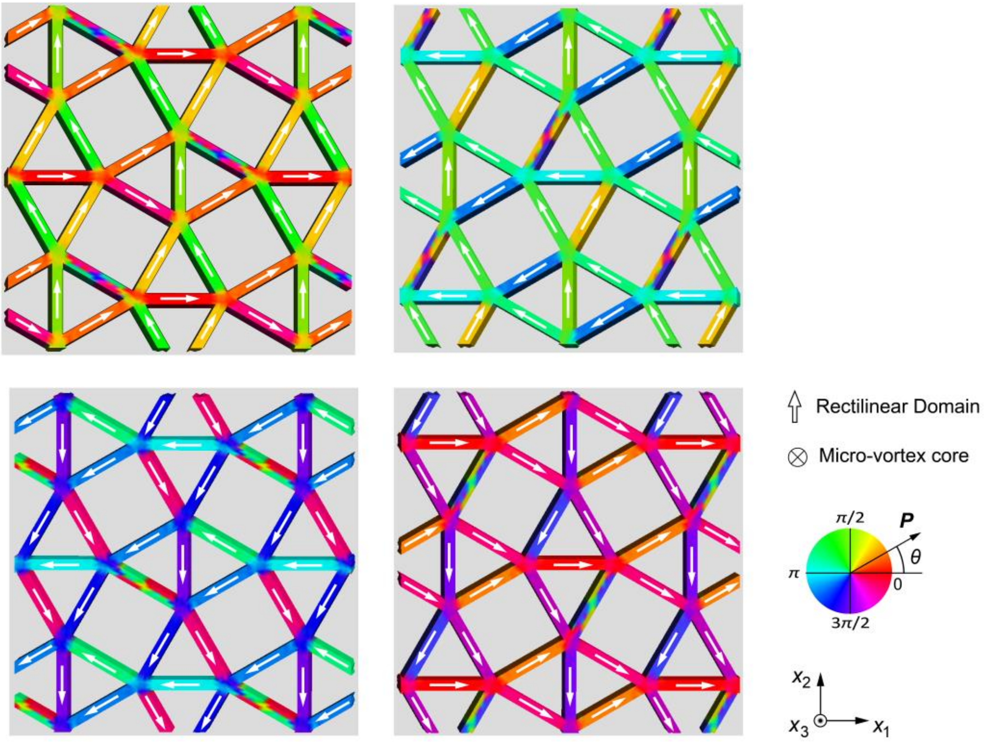

Figure S3(f). 4-equivalent stable states the SrCuBO specimen.

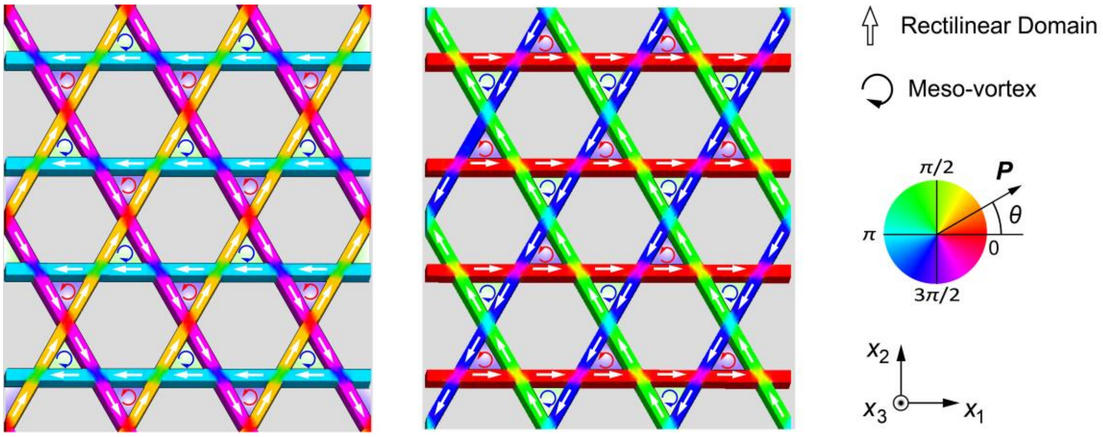

Figure S3(g). 2-equivalent stable states of the Kagome specimen.

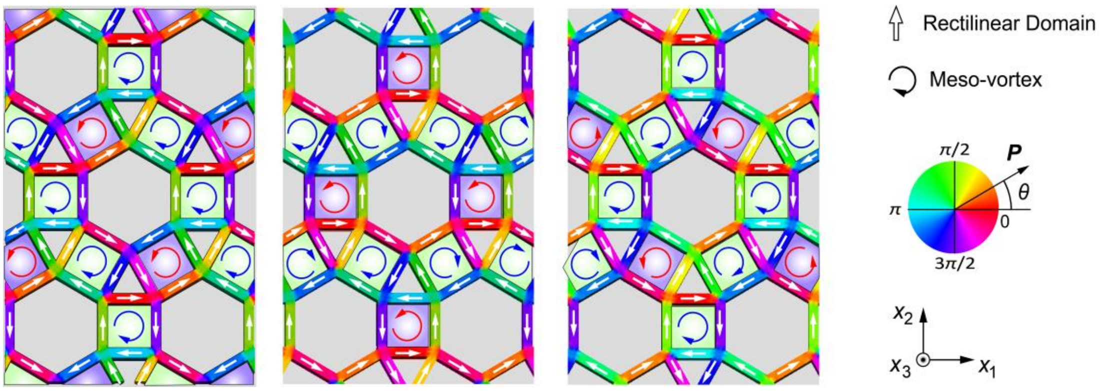

Figure S3(h). 3-equivalent stable states of the Bounce specimen.

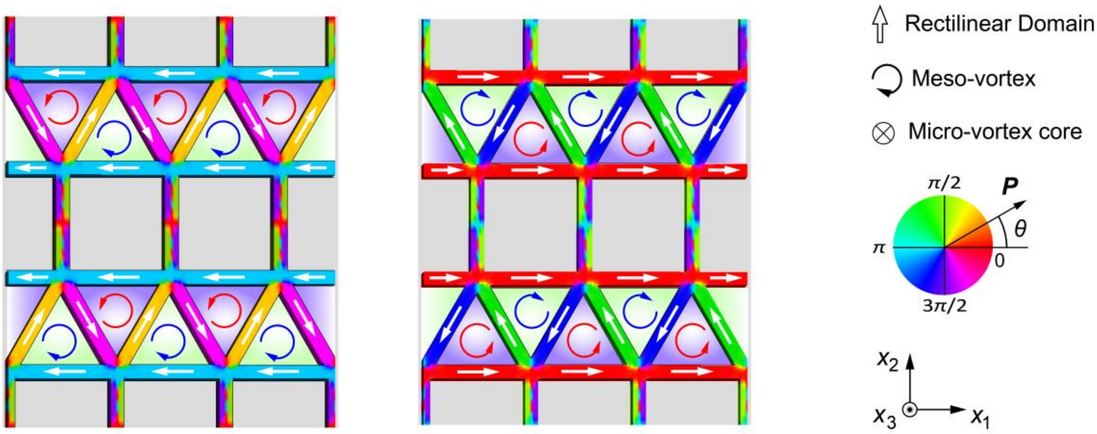

Figure S3(i). 2-equivalent stable states of the Trellis specimen.

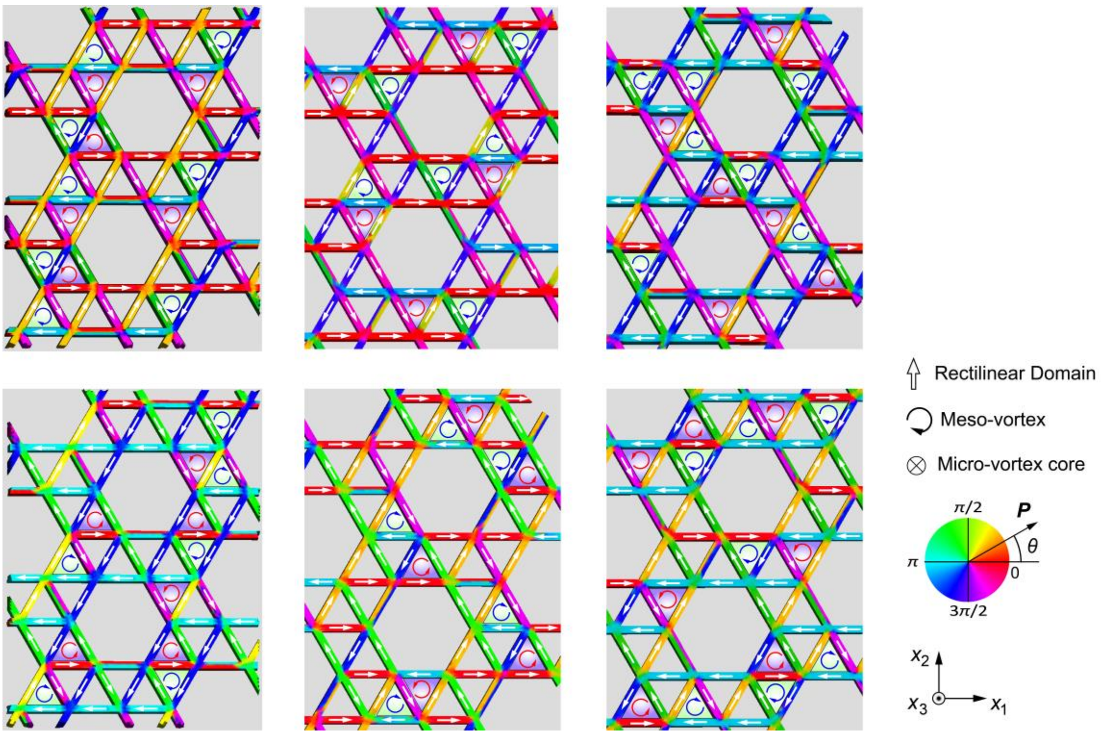

Figure S3(j). 6-equivalent stable states of the Maple-leaf specimen.

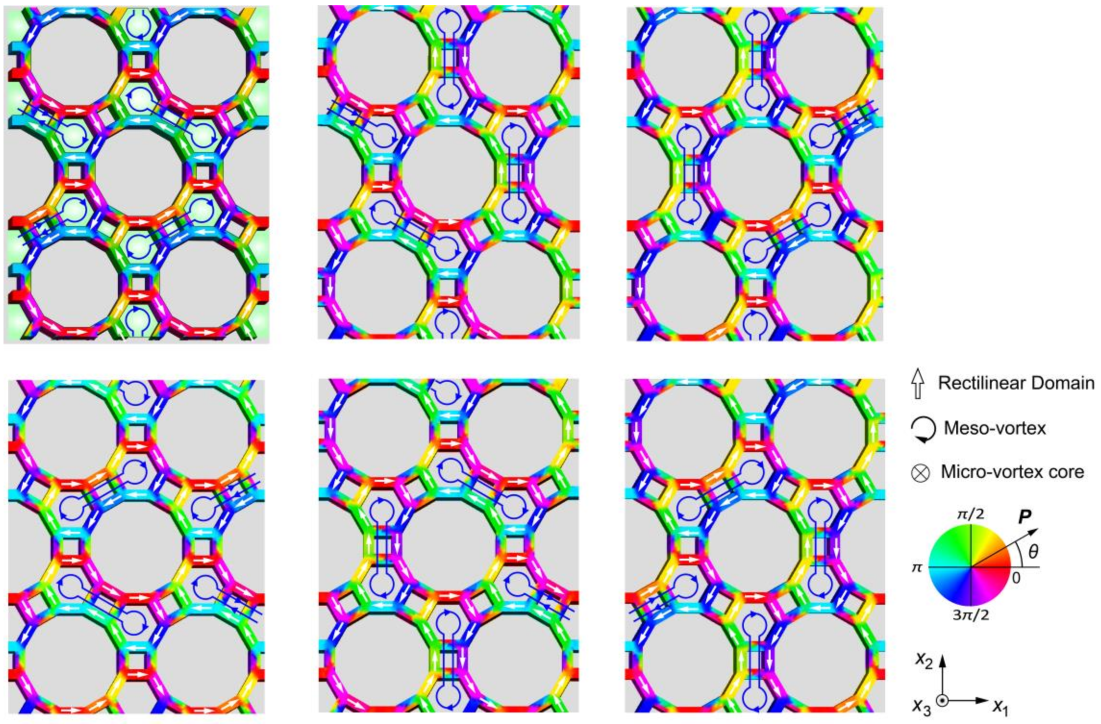

Figure S3(k). 6-equivalent stable states of the SHD specimen.

Figure S3. Equivalent stable states of polarization patterns in ferroelectric nano-metamaterials.

References

[1] Wang, J. & Kamlah, M. Three dimensional finite element modeling of polarization switching in a ferroelectric single domain with an impermeable notch. *Smart Mater. Struct.* **18**, 104008 (2009).

[2] Wang, J. Switching mechanism of polarization vortex in single-crystal ferroelectric nanodots. *Appl. Phys. Lett.* **97**, 192901 (2010).

[3] Wang, J., Kamlah, M., Zhang, T. Y., Li, Y. & Chen, L. Q. Size dependent polarization distribution in ferroelectric nanostructures: Phase field simulations. *Appl. Phys. Lett.* **92**, 162905 (2008).
